# Nobel prize for scientist Professor Robert G Edwards

**DOI:** 10.4103/0974-1208.74151

**Published:** 2010

**Authors:** Kamini Rao

**Affiliations:** Editor-in-Chief, Journal of Human Reproductive Sciences E-mail: kambacc@vsnl.com


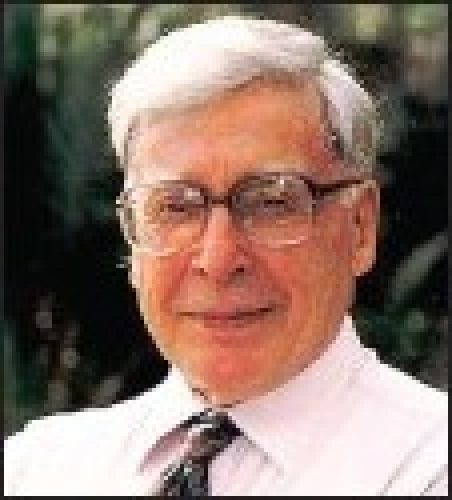


The British physiologist Robert Edwards, whose work led to the first “test-tube baby,” won the 2010 Nobel prize in Medicine for the development of *in vitro* fertilization (IVF), a breakthrough that has helped millions of infertile couples worldwide to have children.

Robert Geoffrey Edwards was born in September 1925. After finishing his primary education at the Manchester Central High School, he served at the University College of North Wales (UCNW) in Bangor, but soon realized that he was interested not so much in plants but rather in animal reproduction and got himself transferred to the Department of Zoology, receiving his B.Sc. in 1951 from UCNW. In 1962, the same institution offered him the degree of D.Sc. After working for 1 year at the California Institute of Technology, he got a 5-year position at the National Institute for Medical Research in London. He then studied at the Institute of Animal Genetics, University of Edinburgh, and received his Ph.D. in 1955. In 1963, he joined Cambridge University. In 1968, he attended a lecture at the Royal Society of Medicine in London given by Patrick Steptoe, a gynecologist, describing laparoscopy, a surgical technique that could give access to the ovaries, enabling the retrieval of eggs in order to be fertilized in vitro. Their collaboration started in 1968 and 10 years later, Louise Brown was born. By 1968, he was able to achieve fertilization of the human egg in the laboratory and started to collaborate with Patrick Steptoe. Edwards and Steptoe established the Bourn Hall Clinic in Cambridge, the world’s first center for IVF therapy. Steptoe was its medical director until his death in 1988 and Edwards was its head of research until his retirement. Gynecologists and cell biologists from all around the world trained at Bourn Hall, where the methods of IVF were continuously refined. Edwards developed human culture media to allow the fertilization and early embryo culture while Steptoe used laparoscopy to recover oocytes from patients with tubal infertility. The birth of Louise Brown, the first “test-tube baby,” in July 1978 heralded the beginning of a new field of medicine.

Robert Edwards was a founder member of ESHRE and became the Society’s first chairman in 1985. The following year, under his drive and direction, ESHRE published the first issue of its journal, Human Reproduction, with him as an Editor, a role he continued for 15 years.

Professor Edwards also founded the journal, “Reproductive Bio Medicine Online” in 2000 and published it independently for 10 years. Having developed it and ensured its place among the leading journals in the field of Reproductive Medicine, he began handing over the reins for the journal in 2009, becoming Editor Emeritus of RBM Online. In 2010, Elsevier became the Publisher of this journal.

The smiles on the face of millions of childless couples worldwide are proof enough of the importance of the discovery of IVF. The artificial reproduction techniques fraternity will unanimously agree that this Nobel prize is well deserved for the courageous breakthrough in infertility, which has now spread from Bourn Hall, UK, to every nook and corner of the world.

ISAR offers its heartfelt congratulations to Professor Edwards on this most distinguished recognition of his achievements in medicine.

